# 4-Meth­oxy-3-(4-nitro­benz­yloxy)benzaldehyde

**DOI:** 10.1107/S1600536811016618

**Published:** 2011-05-11

**Authors:** Zhong-Yu Duan, Guo-Li Ma, Li-Ping Yang

**Affiliations:** a College of Chemical Engineering, Hebei University of Technology, Tianjin 300130, People’s Republic of China

## Abstract

In the title compound, C_15_H_13_NO_5_, the two benzene rings make a dihedral angle of 3.98 (7)°. The crystal packing is stabilized by weak non-classical inter­molecular C—H⋯O inter­actions that link mol­ecules into centrosymmetric tetra­mers.

## Related literature

For general background to the use of Schiff base derivatives in the development protein and enzyme mimics, see: Santos *et al.* (2001 ▶). For a closely related crystal structure, see: Li & Chen (2008 ▶). For reference bond-length data, see: Allen *et al.* (1987 ▶).
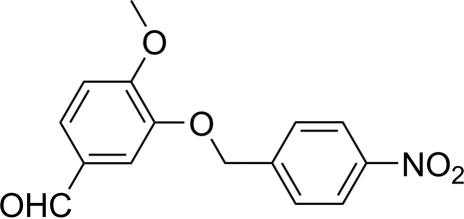


## Experimental

### 

#### Crystal data

C_15_H_13_NO_5_
*M* *_r_* = 287.26
Monoclinic, 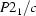

*a* = 6.853 (1) Å
*b* = 11.994 (2) Å
*c* = 16.405 (3) Å
β = 98.28 (3)°
*V* = 1334.4 (4) Å^3^
*Z* = 4
Mo *K*α radiation
μ = 0.11 mm^−1^
*T* = 294 K
0.22 × 0.16 × 0.11 mm

#### Data collection

Bruker SMART APEX CCD area-detector diffractometer
Absorption correction: multi-scan (*SADABS*; Sheldrick, 1996 ▶) *T*_min_ = 0.932, *T*_max_ = 0.988
10078 measured reflections
3161 independent reflections
2441 reflections with *I* > 2σ(*I*)
*R*_int_ = 0.047

#### Refinement

*R*[*F* ^2^ > 2σ(*F* ^2^)] = 0.057
*wR*(*F* ^2^) = 0.133
*S* = 1.12
3161 reflections
191 parameters
H-atom parameters constrained
Δρ_max_ = 0.26 e Å^−3^
Δρ_min_ = −0.21 e Å^−3^

### 

Data collection: *SMART* (Bruker, 1999 ▶); cell refinement: *SAINT* (Bruker, 1999 ▶); data reduction: *SAINT*; program(s) used to solve structure: *SHELXS97* (Sheldrick, 2008 ▶); program(s) used to refine structure: *SHELXL97* (Sheldrick, 2008 ▶); molecular graphics: *SHELXTL* (Sheldrick, 2008 ▶); software used to prepare material for publication: *SHELXTL*.

## Supplementary Material

Crystal structure: contains datablocks I, global. DOI: 10.1107/S1600536811016618/wn2430sup1.cif

Structure factors: contains datablocks I. DOI: 10.1107/S1600536811016618/wn2430Isup2.hkl

Supplementary material file. DOI: 10.1107/S1600536811016618/wn2430Isup3.cml

Additional supplementary materials:  crystallographic information; 3D view; checkCIF report

## Figures and Tables

**Table 1:** Hydrogen-bond geometry (Å, °)

*D*—H⋯*A*	*D*—H	H⋯*A*	*D*⋯*A*	*D*—H⋯*A*
C12—H12⋯O3^i^	0.93	2.42	3.280 (2)	154
C9—H9*A*⋯O5^ii^	0.97	2.53	3.383 (2)	147
C8—H8*B*⋯O4^iii^	0.96	2.55	3.410 (3)	150

Symmetry codes: (i) 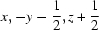; (ii) 
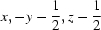
; (iii) 
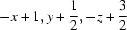
.

**Fig. 1.**
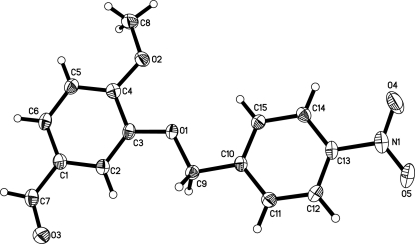
The molecular structure of the title compound, with displacement ellipsoids for non-H atoms drawn at the 50% probability level. Hydrogen atoms are shown as spheres of arbitrary radius.

**Fig. 2.**
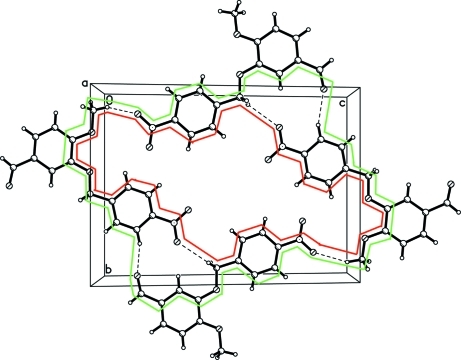
A packing diagram of the crystal structure, with H bonds drawn as dashed lines. The tetramers are indicated by red and green lines.

## supplementary crystallographic information

### Comment

Many Schiff base derivatives have been synthesized and employed to develop protein and enzyme mimics (Santos *et al.*, 2001). The synthesis and crystal structures of numerous derivatives have been published. In particular, the isomeric 3-methoxy-4-(4-nitrobenzyloxy)benzaldehyde crystal structure has been reported (Li & Chen, 2008). As a part of our interest in the coordination properties of Schiff bases functioning as ligands, we have investigated the title compound, which has been used as a precursor in the preparation of Schiff bases.

In the title molecule (Fig. 1), bond lengths (Allen *et al.*, 1987) and angles are within normal ranges. The two benzene rings make a dihedral angle of 3.98 (7)° with each other. A similar value of 4.99 (6)° is observed in 3-methoxy-4-(4-nitrobenzyloxy)benzaldehyde (Li & Chen, 2008).

The crystal packing is stabilised by weak, non-classical intermolecular C12—H12···O3═C7, C8—H8B···O4 and C9—H9A···O5 interactions that link adjacent molecules into centrosymmetric tetramers (Table 1, Fig. 2).

### Experimental

An anhydrous acetonitrile solution (100 ml) of 3-hydroxy-4-methoxybenzaldehyde (1.52 g, 10 mmol) was added dropwise to a solution (50 ml) of 1-(bromomethyl)-4-nitrobenzene (2.16 g, 10 mmol) and pyridine (0.79 g, 10 mmol) in acetonitrile, over a period of 30 min., and the mixture refluxed for 24 h under a nitrogen atmosphere. The solvent was removed and the resultant mixture poured into ice-water (100 ml). The yellow precipitate was then isolated and recrystallized from acetonitrile. It was then dried in a vacuum to give the pure compound in 78% yield. Pale-yellow single crystals of the title compound suitable for X-ray analysis were obtained by slow evaporation of an acetonitrile solution.

### Refinement

The H atoms were included at calculated positions and refined using a riding model approximation. Constrained C—H bond lengths and isotropic U parameters: 0.93 Å and *U*_iso_(H) = 1.2*U*_eq_(C) for C*sp*^2^—H; 0.97 Å and *U*_iso_(H) = 1.2*U*_eq_(C) for methylene C—H; 0.96 Å and *U*_iso_(H) = 1.5*U*_eq_(C) for methyl C—H.

### Crystal data

**Table:**

C_15_H_13_NO_5_	*F*(000) = 600
*M**_r_* = 287.26	*D*_x_ = 1.430 Mg m^−^^3^
Monoclinic, *P*2_1_/*c*	Mo *K*α radiation, λ = 0.71073 Å
Hall symbol: -P 2ybc	Cell parameters from 3519 reflections
*a* = 6.853 (1) Å	θ = 2.3–26.2°
*b* = 11.994 (2) Å	µ = 0.11 mm^−^^1^
*c* = 16.405 (3) Å	*T* = 294 K
β = 98.28 (3)°	Block, pale-yellow
*V* = 1334.4 (4) Å^3^	0.22 × 0.16 × 0.11 mm
*Z* = 4	

### Data collection

**Table:**

Bruker SMART APEX CCD area-detector diffractometer	3161 independent reflections
Radiation source: fine-focus sealed tube	2441 reflections with *I* > 2σ(*I*)
graphite	*R*_int_ = 0.047
φ and ω scans	θ_max_ = 27.9°, θ_min_ = 2.1°
Absorption correction: multi-scan (*SADABS*; Sheldrick, 1996)	*h* = −8→9
*T*_min_ = 0.932, *T*_max_ = 0.988	*k* = −14→15
10078 measured reflections	*l* = −19→21

### Refinement

**Table:**

Refinement on *F*^2^	Primary atom site location: structure-invariant direct methods
Least-squares matrix: full	Secondary atom site location: difference Fourier map
*R*[*F*^2^ > 2σ(*F*^2^)] = 0.057	Hydrogen site location: inferred from neighbouring sites
*wR*(*F*^2^) = 0.133	H-atom parameters constrained
*S* = 1.12	*w* = 1/[σ^2^(*F*_o_^2^) + (0.0561*P*)^2^ + 0.2245*P*] where *P* = (*F*_o_^2^ + 2*F*_c_^2^)/3
3161 reflections	(Δ/σ)_max_ < 0.001
191 parameters	Δρ_max_ = 0.26 e Å^−^^3^
0 restraints	Δρ_min_ = −0.21 e Å^−^^3^

### Special details

**Table:**

Geometry. All e.s.d.'s (except the e.s.d. in the dihedral angle between two l.s. planes) are estimated using the full covariance matrix. The cell e.s.d.'s are taken into account individually in the estimation of e.s.d.'s in distances, angles and torsion angles; correlations between e.s.d.'s in cell parameters are only used when they are defined by crystal symmetry. An approximate (isotropic) treatment of cell e.s.d.'s is used for estimating e.s.d.'s involving l.s. planes.
Refinement. Refinement of *F*^2^ against ALL reflections. The weighted *R*-factor *wR* and goodness of fit *S* are based on *F*^2^, conventional *R*-factors *R* are based on *F*, with *F* set to zero for negative *F*^2^. The threshold expression of *F*^2^ > σ(*F*^2^) is used only for calculating *R*-factors(gt) *etc*. and is not relevant to the choice of reflections for refinement. *R*-factors based on *F*^2^ are statistically about twice as large as those based on *F*, and *R*- factors based on ALL data will be even larger.

### Fractional atomic coordinates and isotropic or equivalent isotropic displacement parameters (Å^2^)

**Table:**

	*x*	*y*	*z*	*U*_iso_*/*U*_eq_	
N1	0.3564 (2)	−0.20291 (14)	0.78771 (10)	0.0282 (4)	
O1	0.23378 (17)	0.07084 (9)	0.45431 (7)	0.0228 (3)	
O2	0.27616 (18)	0.28142 (10)	0.46497 (8)	0.0261 (3)	
O3	0.1082 (2)	−0.00461 (10)	0.13474 (9)	0.0356 (4)	
O4	0.3643 (2)	−0.13342 (13)	0.84335 (9)	0.0419 (4)	
O5	0.3704 (2)	−0.30380 (11)	0.79970 (9)	0.0387 (4)	
C1	0.1427 (2)	0.14338 (14)	0.23296 (11)	0.0216 (4)	
C2	0.1670 (2)	0.07594 (14)	0.30415 (11)	0.0204 (4)	
H2	0.1562	−0.0012	0.2995	0.024*	
C3	0.2070 (2)	0.12507 (14)	0.38043 (11)	0.0199 (4)	
C4	0.2282 (2)	0.24204 (14)	0.38720 (11)	0.0214 (4)	
C5	0.2003 (3)	0.30806 (14)	0.31727 (12)	0.0241 (4)	
H5A	0.2104	0.3852	0.3218	0.029*	
C6	0.1573 (2)	0.25824 (14)	0.24028 (11)	0.0237 (4)	
H6	0.1380	0.3024	0.1932	0.028*	
C7	0.1090 (3)	0.09431 (15)	0.15046 (12)	0.0274 (4)	
H7	0.0862	0.1432	0.1061	0.033*	
C8	0.3346 (3)	0.39608 (15)	0.47346 (13)	0.0326 (5)	
H8A	0.4333	0.4111	0.4389	0.049*	
H8B	0.3874	0.4110	0.5298	0.049*	
H8C	0.2221	0.4430	0.4573	0.049*	
C9	0.2195 (2)	−0.04779 (13)	0.45450 (11)	0.0196 (4)	
H9A	0.3148	−0.0800	0.4229	0.024*	
H9B	0.0886	−0.0709	0.4298	0.024*	
C10	0.2599 (2)	−0.08656 (13)	0.54244 (10)	0.0174 (3)	
C11	0.2435 (2)	−0.19985 (14)	0.55914 (11)	0.0221 (4)	
H11	0.2101	−0.2496	0.5159	0.027*	
C12	0.2765 (2)	−0.23909 (14)	0.63929 (12)	0.0226 (4)	
H12	0.2654	−0.3146	0.6505	0.027*	
C13	0.3264 (2)	−0.16299 (14)	0.70222 (11)	0.0209 (4)	
C14	0.3467 (2)	−0.05042 (14)	0.68780 (11)	0.0210 (4)	
H14	0.3820	−0.0011	0.7312	0.025*	
C15	0.3131 (2)	−0.01262 (14)	0.60716 (11)	0.0198 (4)	
H15	0.3263	0.0629	0.5962	0.024*	

### Atomic displacement parameters (Å^2^)

**Table:**

	*U*^11^	*U*^22^	*U*^33^	*U*^12^	*U*^13^	*U*^23^
N1	0.0235 (7)	0.0388 (9)	0.0219 (9)	−0.0008 (7)	0.0020 (6)	0.0092 (7)
O1	0.0325 (7)	0.0192 (6)	0.0165 (7)	0.0005 (5)	0.0032 (5)	0.0041 (5)
O2	0.0361 (7)	0.0228 (7)	0.0194 (7)	−0.0045 (5)	0.0035 (5)	−0.0001 (5)
O3	0.0508 (9)	0.0282 (7)	0.0270 (8)	−0.0017 (6)	0.0027 (7)	−0.0002 (6)
O4	0.0527 (9)	0.0535 (9)	0.0186 (8)	0.0001 (7)	0.0021 (7)	−0.0008 (7)
O5	0.0439 (8)	0.0390 (8)	0.0325 (9)	0.0001 (6)	0.0031 (6)	0.0191 (7)
C1	0.0198 (8)	0.0251 (9)	0.0200 (9)	−0.0004 (7)	0.0036 (7)	0.0021 (7)
C2	0.0184 (8)	0.0222 (8)	0.0205 (9)	−0.0006 (6)	0.0027 (7)	0.0027 (7)
C3	0.0184 (8)	0.0226 (9)	0.0194 (9)	0.0013 (6)	0.0048 (7)	0.0059 (7)
C4	0.0190 (8)	0.0246 (9)	0.0213 (9)	−0.0002 (6)	0.0051 (7)	0.0005 (7)
C5	0.0269 (9)	0.0210 (8)	0.0247 (10)	0.0006 (7)	0.0044 (7)	0.0041 (7)
C6	0.0239 (8)	0.0253 (9)	0.0221 (10)	0.0025 (7)	0.0045 (7)	0.0081 (7)
C7	0.0303 (10)	0.0307 (10)	0.0211 (10)	0.0016 (8)	0.0037 (7)	0.0060 (8)
C8	0.0448 (11)	0.0232 (9)	0.0286 (11)	−0.0065 (8)	0.0007 (9)	−0.0028 (8)
C9	0.0209 (8)	0.0189 (8)	0.0194 (9)	0.0004 (6)	0.0039 (6)	0.0016 (7)
C10	0.0153 (7)	0.0218 (8)	0.0159 (9)	0.0011 (6)	0.0047 (6)	0.0025 (7)
C11	0.0235 (8)	0.0218 (8)	0.0212 (10)	−0.0009 (7)	0.0037 (7)	−0.0004 (7)
C12	0.0222 (8)	0.0194 (8)	0.0264 (10)	0.0004 (6)	0.0037 (7)	0.0051 (7)
C13	0.0174 (8)	0.0281 (9)	0.0173 (9)	0.0029 (7)	0.0031 (6)	0.0066 (7)
C14	0.0194 (8)	0.0245 (9)	0.0186 (9)	0.0017 (7)	0.0013 (7)	−0.0013 (7)
C15	0.0186 (8)	0.0193 (8)	0.0218 (9)	0.0010 (6)	0.0041 (7)	0.0018 (7)

### Geometric parameters (Å, °)

**Table:**

N1—O5	1.228 (2)	C7—H7	0.9300
N1—O4	1.232 (2)	C8—H8A	0.9600
N1—C13	1.468 (2)	C8—H8B	0.9600
O1—C3	1.365 (2)	C8—H8C	0.9600
O1—C9	1.4262 (19)	C9—C10	1.503 (2)
O2—C4	1.356 (2)	C9—H9A	0.9700
O2—C8	1.433 (2)	C9—H9B	0.9700
O3—C7	1.214 (2)	C10—C15	1.391 (2)
C1—C6	1.385 (2)	C10—C11	1.394 (2)
C1—C2	1.411 (2)	C11—C12	1.384 (2)
C1—C7	1.463 (3)	C11—H11	0.9300
C2—C3	1.374 (2)	C12—C13	1.383 (3)
C2—H2	0.9300	C12—H12	0.9300
C3—C4	1.413 (2)	C13—C14	1.381 (2)
C4—C5	1.384 (2)	C14—C15	1.386 (2)
C5—C6	1.390 (3)	C14—H14	0.9300
C5—H5A	0.9300	C15—H15	0.9300
C6—H6	0.9300		
			
O5—N1—O4	123.67 (17)	H8A—C8—H8B	109.5
O5—N1—C13	118.13 (16)	O2—C8—H8C	109.5
O4—N1—C13	118.20 (15)	H8A—C8—H8C	109.5
C3—O1—C9	118.49 (13)	H8B—C8—H8C	109.5
C4—O2—C8	116.89 (14)	O1—C9—C10	107.92 (13)
C6—C1—C2	120.00 (16)	O1—C9—H9A	110.1
C6—C1—C7	118.69 (16)	C10—C9—H9A	110.1
C2—C1—C7	121.27 (16)	O1—C9—H9B	110.1
C3—C2—C1	119.48 (16)	C10—C9—H9B	110.1
C3—C2—H2	120.3	H9A—C9—H9B	108.4
C1—C2—H2	120.3	C15—C10—C11	119.41 (16)
O1—C3—C2	126.01 (15)	C15—C10—C9	121.81 (15)
O1—C3—C4	113.84 (15)	C11—C10—C9	118.78 (15)
C2—C3—C4	120.13 (16)	C12—C11—C10	120.76 (16)
O2—C4—C5	124.50 (16)	C12—C11—H11	119.6
O2—C4—C3	115.35 (15)	C10—C11—H11	119.6
C5—C4—C3	120.15 (17)	C13—C12—C11	118.26 (16)
C4—C5—C6	119.54 (16)	C13—C12—H12	120.9
C4—C5—H5A	120.2	C11—C12—H12	120.9
C6—C5—H5A	120.2	C14—C13—C12	122.49 (16)
C1—C6—C5	120.64 (16)	C14—C13—N1	118.61 (16)
C1—C6—H6	119.7	C12—C13—N1	118.90 (16)
C5—C6—H6	119.7	C13—C14—C15	118.45 (16)
O3—C7—C1	125.79 (17)	C13—C14—H14	120.8
O3—C7—H7	117.1	C15—C14—H14	120.8
C1—C7—H7	117.1	C14—C15—C10	120.61 (15)
O2—C8—H8A	109.5	C14—C15—H15	119.7
O2—C8—H8B	109.5	C10—C15—H15	119.7

### Hydrogen-bond geometry (Å, °)

**Table:**

*D*—H···*A*	*D*—H	H···*A*	*D*···*A*	*D*—H···*A*
C12—H12···O3^i^	0.93	2.42	3.280 (2)	154
C9—H9A···O5^ii^	0.97	2.53	3.383 (2)	147
C8—H8B···O4^iii^	0.96	2.55	3.410 (3)	150

Symmetry codes: (i) *x*, −*y*−1/2, *z*+1/2; (ii) *x*, −*y*−1/2, *z*−1/2; (iii) −*x*+1, *y*+1/2, −*z*+3/2.
